# Formation of Amyloid-Like Fibrils by Y-Box Binding Protein 1 (YB-1) Is Mediated by Its Cold Shock Domain and Modulated by Disordered Terminal Domains

**DOI:** 10.1371/journal.pone.0036969

**Published:** 2012-05-08

**Authors:** Sergey G. Guryanov, Olga M. Selivanova, Alexey D. Nikulin, Gennady A. Enin, Bogdan S. Melnik, Dmitry A. Kretov, Igor N. Serdyuk, Lev P. Ovchinnikov

**Affiliations:** 1 Group of Protein Biosynthesis Regulation, Institute of Protein Research, Russian Academy of Sciences, Pushchino, Russia; 2 Group of Nucleoprotein Physics, Institute of Protein Research, Russian Academy of Sciences, Pushchino, Russia; 3 Group for Structural Studies of Ribosomal Proteins, Institute of Protein Research, Russian Academy of Sciences, Pushchino, Russia; 4 Laboratory of Protein Physics, Institute of Protein Research, Russian Academy of Sciences, Pushchino, Russia; Russian Academy of Sciences, Institute for Biological Instrumentation, Russian Federation

## Abstract

YB-1, a multifunctional DNA- and RNA-binding nucleocytoplasmic protein, is involved in the majority of DNA- and mRNA-dependent events in the cell. It consists of three structurally different domains: its central cold shock domain has the structure of a β-barrel, while the flanking domains are predicted to be intrinsically disordered. Recently, we showed that YB-1 is capable of forming elongated fibrils under high ionic strength conditions. Here we report that it is the cold shock domain that is responsible for formation of YB-1 fibrils, while the terminal domains differentially modulate this process depending on salt conditions. We demonstrate that YB-1 fibrils have amyloid-like features, including affinity for specific dyes and a typical X-ray diffraction pattern, and that in contrast to most of amyloids, they disassemble under nearly physiological conditions.

## Introduction

YB-1 is a multifunctional nucleocytoplasmic protein that interacts with both RNA and DNA. YB-1 mediates mRNA localization on tubulin [1], [2] and actin [3] cytoskeletons. It can be found in stress granules [4], P-bodies [5] and centrosomes [6], [7]. YB-1 is involved in regulation of transcription [8], [9] and translation [10], [11], alternative splicing [12], [13], DNA reparation, gene-toxic stress response, and development of multidrug resistance [14]. Depending on its amount, intracellular localization, and cell context, YB-1 may act either as an antioncoprotein [15], [16] or an oncoprotein [17], stimulate the epithelial-mesenchymal transition and metastasis [18], and serve as a marker of cancer cells and their aggressiveness [19], [20]. Recently, it was shown that YB-1 can be secreted by a non-classical mechanism [21]. Extracellular YB-1 stimulates migration and proliferation of mesangial cells [21] through activation of Notch-3 receptors [22]. For a comprehensive review of YB-1 functions, see [23].

Mammalian YB-1 consists of a five-stranded β-barrel-structured cold shock domain (CSD) [24] flanked by N- and C-terminal domains. The N-terminal domain is known as an A/P-domain because it is rich in alanine and proline, and the C-terminal domain contains alternating clusters of positively and negatively charged amino acid residues. The tertiary structure of the terminal domains remains unknown and is believed to be disordered. As shown previously, in solution and in protein-saturated complexes with mRNA, YB-1 associates in multimers up to 800 кDa [25], [26]. Recently, we reported that YB-1 formed fibrils under high ionic strength conditions, e. g., in the presence of 2 M LiCl. These fibrils were 15 nm wide, had the shape of a helix with a period of 52 nm, and their ends showed polarity. It was conjectured that YB-1 fibrils were amyloids by nature [27].

Amyloids were discovered as protein deposits associated with several neurodegenerative diseases including Alzheimer's disease [28]. They have elongated, unbranched fibril morphology, display affinity for Congo red and Thioflavin T (ThT), and give a cross-β X-ray diffraction pattern [29]. A number of proteins form *in vitro* similar structures termed amyloid-like [28]. Later, several proteins were reported to form functional structures with amyloid properties [30]: for example, peptide hormones can be secreted in the form of amyloids [31].

Here we describe (i) contribution of each domain of YB-1 to fibril formation, (ii) the salt effect on formation of YB-1 fibrils and the possibility of their appearance at physiological ionic strength, and (iii) properties of the formed YB-1 fibrils. We found that fibril formation by YB-1 is mediated by its CSD, while different regions of its disordered terminal domains differentially contribute to the process. We have shown that YB-1 fibrils are amyloid-like, but this structure is reversible and disassembles under physiological conditions.

## Results

### The cold shock domain of YB-1 is responsible for fibril formation under high ionic strength conditions, whereas its N- and C-terminal domains modulate this process

**Figure 1:**
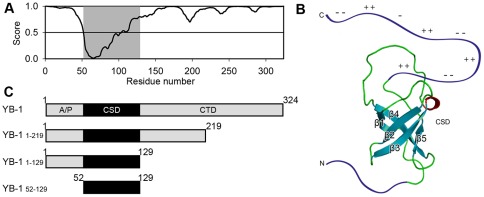
The domain organization of YB-1. (A), Prediction of structured and intrinsically disordered regions of YB-1 by IsUnstruct algorithm. Disorder is assigned to score values greater than or equal to 0.5. CSD is highlighted by gray shading. (B), The tertiary structure of CSD and sketched terminal domains. (C), YB-1 and its fragments used in the study. The indicated regions belong to different YB-1 domains.

YB-1 consists of a conserved β-barrel-structured cold shock domain flanked by N- and C-terminal domains that are predicted to be intrinsically disordered, as shown by the disorder prediction algorithm IsUnstruct [32] (Fig 1A). This prediction is supported by the following two facts. First, 36 kDa YB-1 possesses an abnormal mobility in SDS polyacrylamide gel electrophoresis that corresponds to the mobility of a 50 kDa protein. Second, under physiological conditions, YB-1 shows a circular dichroism spectrum that is characteristic for an unfolded protein (see below). Figure 1B shows the structural organization of YB-1 CSD as follows from NMR [24]. The terminal domains are sketched.

**Figure 2:**
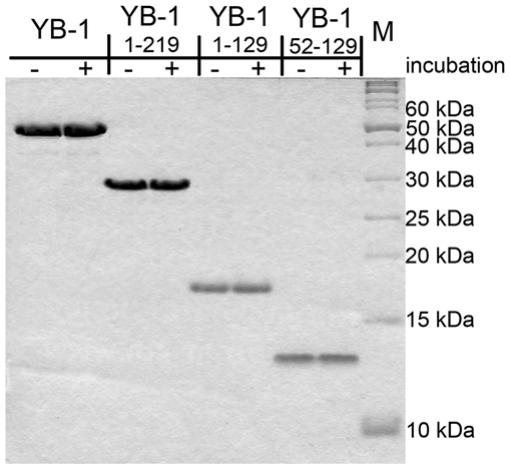
Electrophoretic analysis of YB-1 and its fragments before and after incubation. YB-1 and its fragments before (−) and after (+) 24 h incubation in 2 M LiCl were subjected to 13% SDS-PAGE in a tris-tricine buffer system and stained with Coomassie brilliant blue. Protein molecular weight markers are shown (lane M).

**Figure 3:**
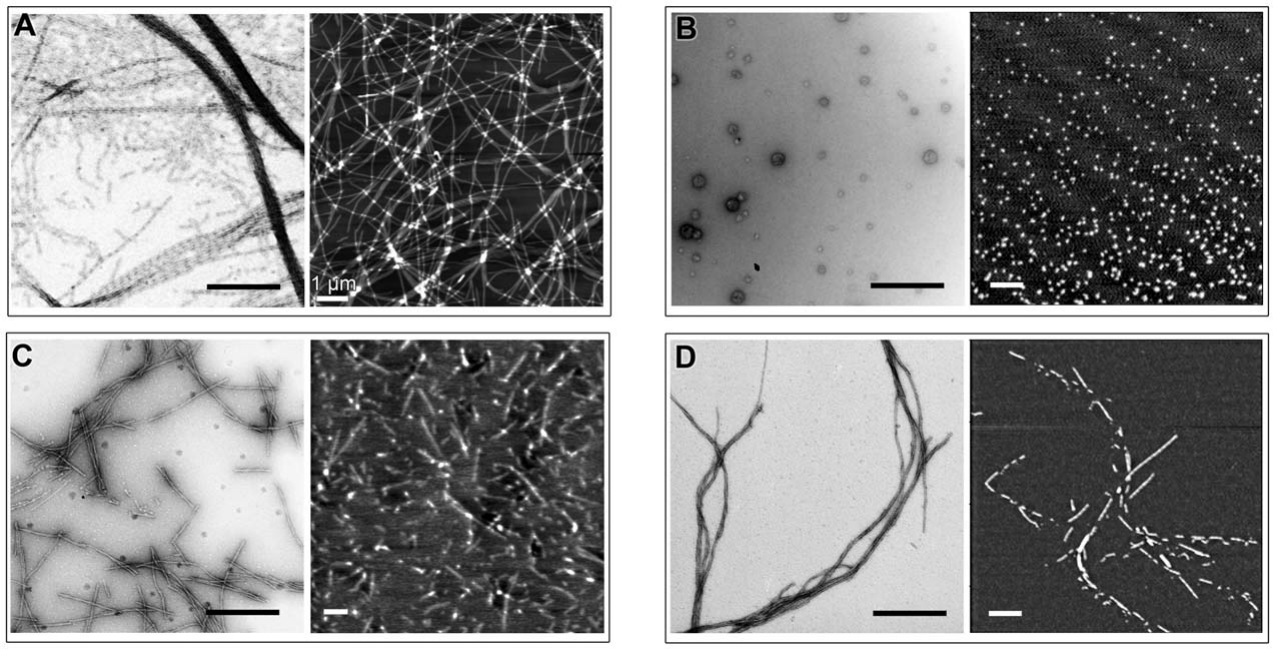
Fibril formation by YB-1 and its fragments. Protein samples (10 µM) were incubated in the presence of 2 M LiCl for 24 h. Supramolecular complex formation was visualized by EM (left) and AFM (right) imaging. (A), YB-1; (B), YB-1_1–219_; (C), YB-1_1–129_; (D), YB-1_52–129_. Scale bars are 0.4 µm where not indicated.

Previously, we reported that YB-1 formed fibrils in the presence of 2 M LiCl [27]. To find out which domain of YB-1 is responsible for fibril formation, we used, apart from the full-length YB-1, its three fragments: (i) YB-1_1–219_ comprising the N-terminal domain, CSD, and the first half of the C-terminal domain. Such a fragment can appear *in vivo* under conditions of a DNA-damaging stress as a result of YB-1 cleavage with 20 S proteasome [33]. It lacks the cytoplasmic retention signal but preserves the nuclear localization site. Therefore, it is mostly located in the nucleus; (ii) YB-1_1–129_ comprising the N-terminal domain and CSD; (iii) YB-1_52–129_ comprising solely CSD. YB-1 and its fragments are schematically shown in Figure 1C. All these samples were incubated with 2 M LiCl for 24 h. The absence of their degradation after incubation was monitored by SDS gel electrophoresis (Fig 2). Then the negatively stained samples were examined by transmission electron microscopy (EM) and atomic force microscopy (AFM) (Figs 3A–D). As found, the full length YB-1 formed fibrils of various length that tended to associate in bundles (Fig 3A), which was consistent with our previous data. The fragment YB-1_1–219_ formed nothing but globular particles (Fig 3B) similar to those described earlier for the full-length YB-1 [26]. YB-1_1–129_ formed short fibrils (Fig 3C) of the same morphology as those formed by the full-length YB-1. YB-1_52–129_ formed long fibrils, although in less abundance (Fig 3D).

This allows concluding that CSD is responsible for YB-1 fibril formation. The N-terminal domain presumably stimulates initiation of this process, as follows from a higher number and a shorter length of the fibrils formed by YB-1_1–129_, as compared to YB-1_52–129_. As to the C-terminal domain, its first half prevents fibril formation, while its last half neutralizes this negative effect.

### Some YB-1 fragments may form fibrils at physiological ionic strength

**Figure 4:**
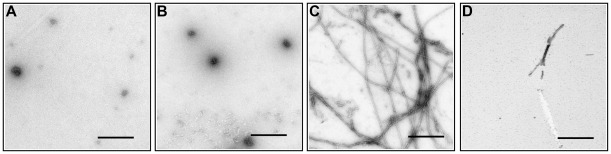
Fibril formation by YB-1 and its fragments under physiological conditions. EM images of YB-1 (A), YB-1_1–219_ (B), YB-1_1–129_ (C), and YB-1_52–129_ (D) (10 µM) incubated in the presence of 0.15 M KCl for 92 h. Scale bars are 0.4 µm.

Next, we learned whether YB-1 and its fragments were capable of forming fibrils at physiological ionic strength. The samples were incubated in the presence of 0.15 M KCl and analyzed by EM imaging (Fig 4). Under these conditions, the full-length YB-1 and YB-1_1–219_ gave merely globular particles (Figs 4A, B), while YB-1_1–129_ formed fibrils as abundantly as at high ionic strength (Fig 4C). With 0.15 M KCl present, YB-1_52–129_ gave a much lower number of fibrils in comparison with that observed in the 2 M LiCl conditions (cf. Fig 4D and Fig 3D). Its ability to form fibrils in physiological conditions was seemingly exceeded by YB-1_1–129_, as shown by microscopy that revealed only rather short sparse fibrils (cf. Fig 4D and Fig 4C). Noteworthily, an increased concentration of YB-1_52–129_ and its incubation for 2 weeks gave elongated fibrils capable of sheet forming (data not shown). Thus, YB-1 CSD can form fibrils at physiological ionic strength as well, and this process is suppressed by the C-terminal domain and stimulated by the N-terminal domain.

### YB-1 fibrils exhibit amyloid-like properties

**Figure 5:**
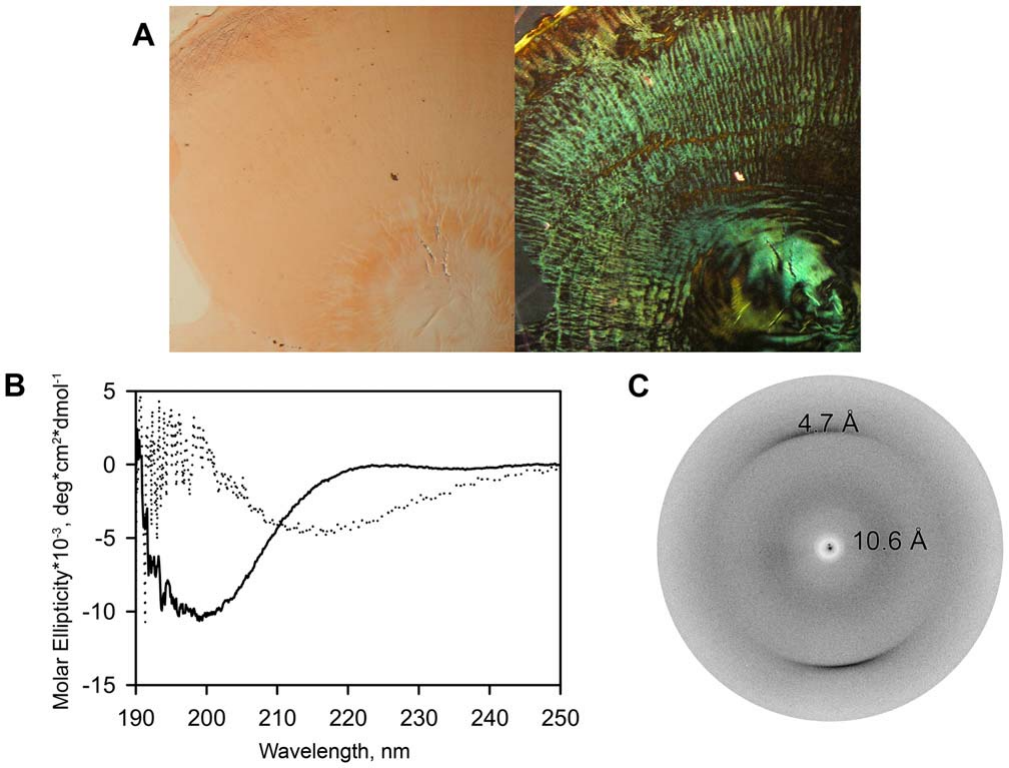
YB-1 forms amyloid-like fibrils. (A), Congo red staining of YB-1 fibrils. YB-1 (∼1.4 mM) was incubated under high ionic strength conditions (2 M LiCl) for 72 h. Photographs were taken in a bright-field mode (left) or under cross-polarized light (right). (B), Circular dichroism spectroscopy of YB-1. Spectra were obtained for 30 µM YB-1 in 0.15 M KCl (solid line) and after incubation with 1 M MgSO_4_ (dotted line). (C), X-ray diffraction of an oriented YB-1 fibril sample.

We presumed that YB-1 fibrils were amyloid-like. Originally, amyloids were recognized by specific staining with Congo red. We incubated concentrated YB-1 in the presence of 2 M LiCl until the protein gel formed. Its staining with Congo red gave green birefringence (Fig 5A) which strongly suggested the amyloid-like nature of YB-1 fibrils.

The main characteristic feature of amyloids is occurrence of the cross-β-structure. We incubated YB-1 under high ionic strength conditions and analyzed changes occurring in its secondary structure using circular dichroism spectrometry. To avoid significant absorption in the far UV region by chloride ions, LiCl was substituted by MgSO_4_. Under physiological conditions, YB-1 had a spectrum with the minimum near 200 nm, which is characteristic for unfolded proteins. Incubation under high salt conditions resulted in a spectrum with the minimum near 217 nm characteristic for the β-structure (Fig 5B). To conclusively prove the formation of the cross-β-structure, we prepared an oriented YB-1 fiber sample and subjected it to X-ray diffraction analysis. Two reflections at ∼4.7 Å and ∼10.6 Å were consistent with the cross-β-sheet structure [34] (Fig 5C). Moreover, after incubation with 0.2 M KCl, YB-1_1–129_ showed the same Congo red staining and X-ray diffraction pattern (data not shown) as those described for YB-1 in 2 M LiCl.

### Effect of different cations on formation of YB-1 amyloid-like fibrils

**Figure 6:**
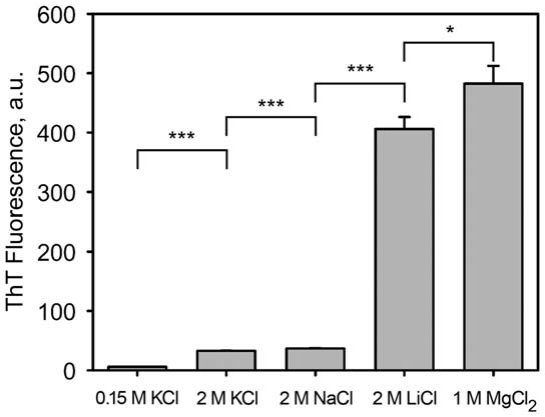
Effect of different salts on YB-1 fibril formation. YB-1 (10 µM) was incubated in 20 mM Hepes-KOH, pH 7.4, containing different salts as indicated under the bars for 24 h at 20°C. Amyloid formation was assessed by ThT fluorescence. Means and SD are shown (n = 3), and *or *** indicates *t*-test *p*<0.05 or *p* <0.001, respectively.

To study the effect of different cations on formation of amyloid-like fibrils by the full-length YB-1, we compared the effects of different salts, namely, KCl, LiCl, MgCl_2_, and NaCl (containing different cations and the same counterion Cl^−^), on this process. YB-1 samples were incubated at high concentration of different metal chlorides, and amyloid formation was assessed from their binding to a specific fluorescent dye Thioflavin T (Fig 6). According to their ability to cause amyloid formation, the cations were ordered Mg^2+^>Li^+^>Na^+^≥K^+^.

### YB-1 fibrils may disassemble under physiological ionic strength conditions

As a rule, the amyloid-like structures show high stability and prove to be resistant even to dissociation with sodium dodecyl sulfate [35].

**Figure 7:**
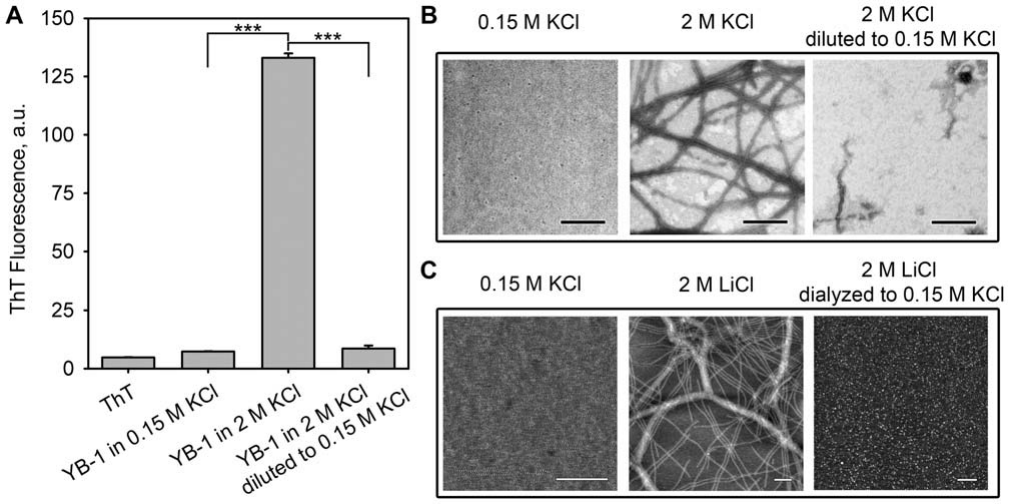
Disassembly of YB-1 amyloid-like fibrils at physiological ionic strength. (A) and (B), YB-1 (56.8 µM) was incubated with 0.15 M or 2 M KCl for 24 h. An aliquot of YB-1 pre-incubated with 2 M KCl was diluted to 0.15 M KCl. Remaining samples were diluted to the same final protein concentration (4.26 µM) with appropriate KCl solutions to keep the salt concentration unchanged. The samples were incubated for 1 h at room temperature and analyzed by (A), ThT fluorescence (means and SD are shown (n = 3), and *** indicates *t*-test *p*<0.001) and (B), EM imaging. (C), YB-1 (30 µM) was incubated with 0.15 M KCl or 2 M LiCl for 24 h. An aliquot of YB-1 pre-incubated with 2 M LiCl was dialyzed against 0.15 M KCl. Fibril formation was visualized by AFM imaging. Scale bars are 0.4 µm. Ionic strength conditions during the incubation are indicated.

It was of interest to learn whether YB-1 fibrils formed at high ionic strength retained their stability at physiological salt concentration. To do so, we grew fibrils in the presence of 2 M KCl and then diluted the sample with water to a final KCl concentration of 0.15 M. In control, YB-1 was incubated with either 0.15 M KCl or 2 M KCl, and then the samples were diluted with 0.15 M KCl or 2 M KCl, respectively, to adjust them to the same protein concentration as in experiment. The presence of amyloid-like aggregates was monitored by ThT fluorescence and EM imaging (Fig 7). As seen from Figure 7A, ThT fluorescence intensity of YB-1 incubated with 0.15 M KCl did not differ much from that of ThT itself. In contrast, YB-1 incubation with 2 M KCl resulted in a markedly increased ThT fluorescence, which indicated the presence of amyloids. Surprisingly, the subsequent decrease in KCl concentration down to 0.15 M reduced ThT fluorescence of YB-1 incubated with 2 M KCl almost to the value shown by the sample after its incubation with 0.15 M KCl. This dramatic drop in ThT fluorescence indicated that YB-1 amyloid-like fibrils had been disassembled.

An analysis of the same samples by EM imaging revealed a good agreement with the ThT results. YB-1 incubation with 0.15 M KCl gave no amyloids (Fig 7B, *left*), whereas its incubation with 2 M KCl resulted in distinct elongated fibrils (Fig 7B, *middle*). The subsequent incubation of such fibrils with 0.15 M KCl made them much shorter and less abundant (Fig 7B, *right*). A similar behavior was demonstrated by YB-1 fibrils grown in 2 M LiCl after substitution of 0.15 M KCl for 2 M LiCl by dialysis (Fig 7C). Thus, amyloid-like fibrils formed by the full-length YB-1 at high ionic strength are able to disintegrate under physiological ionic strength conditions.

## Discussion

We showed previously [27] that the full-length YB-1 forms fibrils at a high concentration of LiCl (2 M), which brought the properties of these fibrils in the focus of our study. As known, in the cell there are fibrillar structures vital for its living, like elements of the cell skeleton that perform structural and transport functions. A distinguishing feature of these structures is their dynamic nature. Alongside with them, there are fibrillar structures termed amyloids that complicate the organism's life by provoking a number of diseases. Amyloids are insoluble protein aggregates produced by some soluble proteins. Their association may occur *in vitro* and involve proteins of various structures. The association-favorable conditions are usually other than physiological, and aggregation is generally irreversible. Fibrillar aggregates formed *in vitro* have been named amyloid-like structures [28], [36]. The characteristics of amyloids and amyloid-like structures are their affinity for Congo red with resulting green birefringence and an X-ray diffraction pattern typical for the cross-β-structure [37].

Previously, the general morphology of YB-1 fibrils, as shown by microscopy, allowed their preliminary assignment to amyloid-like fibrils [27]. In this study we have demonstrated that YB-1 fibrils show the following amyloid properties: (i) ability to be stained with Congo red and Thioflavin T, (ii) an elevated level of the β-structure (as shown by CD spectroscopy), and (iii) a characteristic pattern of X-ray diffraction. However, unlike most of known amyloid-like structures that associate either at extreme pH values or at elevated temperature, YB-1 fibrils are formed at physiological pH values and 20–37°C. The basic prerequisite to YB-1 fibril growth is a high salt concentration. Besides, YB-1 fibrils appear to be reversible and easily disintegrating under conditions of physiological salt concentration. These peculiarities make YB-1 fibrils an interesting subject for further studies.

The stimulating effect of a high salt concentration on formation of amyloid fibrils may occur due to three principal factors: (i) Debye-Hückel screening, (ii) specific ion-peptide interactions, and (iii) changes in the water structure. Generally, Debye-Hückel screening is not considered to be a possible stimulating mechanism of amyloid aggregation [38]–[40]. As reported [40], formation of *β*_2_-microglobulin fibrils is mostly stimulated by the mechanism of specific ion-peptide interactions. Yet, as seen from their effect on α-synuclein amyloid aggregation, the ions are ordered as the Hofmeister series, which points to importance of changes in the general water structure [39]. In case of Aβ(1–40) peptide fibrils, the both mechanisms are presumably involved [38]. According to their ability to cause YB-1 amyloid formation, the cations are ordered Mg^2+^>Li^+^>Na^+^≥K^+^, which coincides with their order for the Aβ(1–40) peptide [38]. It may be believed that the salt ions play a role in YB-1 fibril formation due to both their specific interactions with charged groups of the protein and changes in the water structure.

To identify the YB-1 site responsible for fibril formation, we checked the ability of the studied YB-1 fragments to form fibrils in 2 M LiCl. Similar to the full-length YB-1, this ability was shown by YB-1_1–129_ and YB-1_52–129_, but not by the largest fragment YB-1_1–219_. These results suggest that YB-1 forms fibrils using its CSD, while the first half of the C-terminal domain inhibits the process, and the last half neutralizes this negative effect. The N-terminal domain presumably stimulates initiation of fibril formation, because in its absence YB-1_52–129_ forms a small number of long fibrils, while in its presence the full-length YB-1 and YB-1_1–129_ form many fibrils of various lengths.

At physiological ionic strength the full-length YB-1 and YB-1_1–219_ are unable to form fibrils, while the isolated CSD (YB-1_52–129_) preserves this ability, though the fibrils are formed in less abundance than at high ionic strength. Importantly, the presence of the N-terminal domain enhances the fibril-forming activity of CSD. The first half of the C-terminal domain behaves likewise at both physiological and high ionic strength, i.e., prevents fibril formation, but its last half is ionic strength-dependent: at high ionic strength it neutralizes the inhibiting effect of the first part, while in physiological conditions it does not.

Thus, we have shown that CSD plays the key role in formation of YB-1 amyloid-like fibrils, while the N- and C-terminal domains positively or negatively modulate this process.

To date, many models of amyloid formation have been proposed [29]. It is believed that this process is mediated by protein molecules in a partially unfolded state. Interestingly, the isolated CSD in solution has low stability, and a high percentage of CSD molecules are in the unfolded state [24]. The YB-1 CSD-mediated mechanism of amyloid-like structure formation may be similar to the mechanism of fibril formation by the major cold shock protein *Ec*-CspA from *Escherichia coli*. The sequence identity of this bacterial protein to YB-1 CSD is 47%, and it has a β-barrel spatial structure as well. It was shown that *Ec*-CspA forms amyloid-like fibrils at low pH. As demonstrated by NMR, in these conditions its β-barrel structure is partially unfolded but still preserves some features of the native structure formed by the strands β1β2β3. This suggests that the amyloid cross-β-structure results from intermolecular parallel pairing of the strands β1 and β3 [41].

Another bacterial cold shock protein *Bc*-Csp from *Bacillus caldolyticus* forms domain-swapped dimers when crystallized in complex with hexathymidine [42]. This mechanism may underlie amyloid formation as well, as has been demonstrated for the β-structured protein cystatin C [43]. It cannot be ruled out that the domain swapping mechanism [29], [44] is also used in formation of amyloid-like structures by CSD-containing proteins, including YB-1.

In this study we have shown that the evolutionally added N- and C-terminal domains of YB-1 can significantly influence the CSD ability to form fibrils. It follows from the above results that the charged residue-rich C-terminal domain includes sites producing differential effects on fibril formation. It was previously reported that at physiological ionic strength YB-1 can form multimers through its C-terminal domain [25], [45] that acts like a charged zipper [46]. Another supposition was that in the full-length YB-1 the last half of its C-terminal domain interacts with the first half to give a hairpin-loop structure [33], where the majority of charged residues are presumably involved in interactions with one another, while only few interact with CSD. A high ionic strength possibly prevents this interaction, thereby depriving the C-terminal domain of any influence on the process of fibril formation in these conditions. That is why the full-length YB-1 and YB-1_1–129_ lacking this domain form fibrils with equal efficiency at 2 M LiCl. Then, the absence of YB-1 fibrils at 0.15 M KCl ionic strength may be explained by additional interactions occurring between the hairpin-loop structure and CSD which stabilize the CSD structure and prevent CSD-mediated fibril formation by the full-length YB-1.

With the absent last half of the C-terminal domain, YB-1_1–219_ forms no hairpin-loop structure, and unbound charged residues of the first half can interact with CSD at both low and high ionic strength, thereby preventing CSD-mediated fibril formation. This model is supported by the fact that no fibril formed by YB-1_1–219_ was observed either at low or high ionic strength. With the completely cut off C-terminal domain, stability of CSD in YB-1_1–129_ appears unsupported, and then the CSD-mediated fibril formation becomes independent of ionic conditions. The removal of the N-terminal domain from YB-1_1–129_ makes the fibril-forming ability of the remaining CSD (YB-1_52–129_) much lower at physiological ionic strength. Presumably, the N-terminal domain either promotes CSD transition to a conformation optimal for fibril forming or stabilizes the formed amyloids.

Amyloids have long been considered to be insoluble protein aggregates that can accumulate in an organism and provoke various disfunctions. High stability was believed to be one of their basic features manifested as their resistance against proteolysis with proteinase K [47] or dissociation with sodium dodecyl sulfate [35]. Later, it was found that amyloids could play a physiological role in various organisms, specifically in mammals. For example, an amyloid derived from the protein Pmel17 showed template activity and accelerated covalent polymerization of reactive molecules into melanin [48]. Interestingly, the repeat domain of Pmel17 formed amyloids under mildly acidic conditions, while under neutral conditions these amyloids dissolved [49]. Moreover, peptide and protein hormones in secretory granules of the endocrine system can be stored in an amyloid-like cross-β-sheet-rich conformation. Upon signaling, secretory granules are secreted, and the cross-β-sheet structure of the amyloid enables a controlled release of monomeric, functional hormone [31].

Among the studied conditions, only high ionic strength allows efficient fibril formation by the full-length YB-1. However, it cannot be ruled out that the complex intracellular environment can produce conditions favorable for formation of amyloid-like structures by YB-1, e.g., in the course of its post-translational modifications or its interactions with ligands. It is known that YB-1 is phosphorylated by some kinases and binds not only to DNA and mRNA but also to many proteins. The literature describes the effect of ligands on fibril formation. For example, some peptide hormones form amyloid structures exclusively in the presence of glycosaminoglycans [31].

When in complex with ligands, YB-1 can be a component of various intracellular organelles, like stress granules [4] and processing bodies [5], secretory granules [21], centrosomes [6], [7] and nucleoli [50]; in the nucleoplasm YB-1 can show speckled distribution and be a member of so far unidentified structures and complexes [51]; it can interact with microfilaments [3] and microtubules [2], [6] and contribute to formation of the latter [2]. Within these structures YB-1 presumably could realize its ability to form reversible amyloids which might be localized in secretory granules during YB-1 secretion from the cell and then be released into the intercellular space in the way described for peptide hormones [31]. Noteworthily, the macrophage migration inhibitory factor secreted like YB-1 by a non-classical mechanism [52] can form amyloid-like fibrils. But these are effectively formed at pH <4, and their ability to dissociate in physiological conditions has not been assayed [53].

Apart from the above, the amyloid-like conformation can serve for YB-1 storing in the cell.

It may be expected that in the cell fibrils can be formed by YB-1 fragments. As shown in the current study, fibril formation is effectively performed by some YB-1 fragments in physiological conditions. These fragments are sometimes involved in secretion. For example, a YB-1 fragment with an electrophoretic mobility of about 18 kDa (YB-1/p18) has been recently detected in human blood [54]. This fragment contained CSD but lacked some portions of the N- and C-terminal sequences. Since YB-1/p18 was often detected in plasma of patients with advanced cancerous diseases, it was proposed for the use as a cancer marker [54]. It should be noted that electrophoretic mobility of YB-1/p18 is characteristic for the fragment YB-1_1–129_ that contains CSD (Fig 3) and is able, according to this study, to form fibrils at physiological ionic strength. In contrast, other YB-1 fragments, e.g., YB-1_1–219_ detected in the cell nucleus, cannot form amyloid-like structures in any of the studied conditions, which may also be physiologically relevant.

## Materials and Methods

### Protein purification and fibrillogenesis

The full-length YB-1 was purified as described earlier [27], [55]. Briefly, YB-1 was synthesized in *E. coli* from plasmid pET-3-1-YB-1 and purified by Heparine-Sepharose, MonoS and Superose 12 (GE Healthcare, Sweden) chromatography. Fractions containing pure YB-1 were concentrated and dialyzed against 20 mM Hepes-KOH, 0.5 M KCl, pH 7.4. Plasmid pET-3-1-YB-1_1–219_ was provided by Dr. Alexey Sorokin. YB-1_1–219_ was purified in the same way as YB-1 except that dialyzed against 20 mM Hepes-KOH, 0.15 M KCl, pH 7.4. To construct plasmids for YB-1_1–129_ and YB-1_52–129_, the corresponding DNA fragments were PCR-amplified and inserted into the pET-22b vector. YB-1_1–129_ and YB-1_52–129_ were expressed in *E. coli* and isolated by ammonium sulfate fractionation and chromatography using SP-Sepharose, Phenyl-Sepharose and MonoS columns (GE Healthcare). YB-1_1–129_ and YB-1_52–129_ were stored in 20 mM Hepes-KOH, 0.2 M KCl, pH 7.4 or 50 mM potassium phosphate, pH 7.4, respectively. Concentrations were estimated from 280 nm absorbance using calculated extinction coefficients (ProtParam tool, http://expasy.org/tools/protparam.html). The OD_280_/OD_260_ ratios were ∼2, thus showing the absence of nucleic acids from the protein samples. To test fibril forming capability, the protein samples (10 µM) were incubated in 20 mM Hepes-KOH, pH 7.4, containing 0.15 M KCl for 92 h or 2 M LiCl for 24 h at 20°C and analyzed by EM and AFM.

### Congo red staining

Congo red staining was performed as described [37] with minor modifications. YB-1 (∼1.4 mM or ∼50 mg/ml) was incubated with 2 M LiCl at 20°C. The protein formed gel after 72 h incubation. An aliquot of YB-1 gel was transferred to deionized water (1 ml) for 1 min to wash out the salt, centrifuged at 12,000 rpm and applied onto a glass slide. The air-dried protein spot was stained with 0.1% Congo red in water and photographed using a microscope equipped with two polarizers.

### X-ray diffraction study

An oriented YB-1 fiber sample was prepared using the simplified stretch frame method [56]. The protein gel sample was washed with water as described under **Congo red staining** and placed between waxed ends of glass capillaries mounted in a petri dish. The rod-like air-dried sample was placed under an X-ray beam. The fiber diffraction images were collected using a Microstar X-ray generator with HELIOX optics equipped with a Platinum^135^ CCD detector (X8 Proteum system, Bruker AXS). Cu Kα radiation (λ = 1.54Å) was used. The samples were oriented perpendicular to the X-ray beam using a 4-axis kappa goniometer.

### Transmission EM

Protein samples were diluted to 3 µM of protein, adsorbed onto copper grids coated with 0.2% formvar in chloroform for 2 min, briefly washed with water, stained with 1% uranyl acetate or 2% ammonium molybdate (aqueous solutions) for 1 min, and examined using a JEM-100C microscope (JEOL, Japan) operating at 80 kV.

### AFM imaging

For AFM studies, the protein sample was diluted to 3 µM, and immediately a 5 µl aliquot was applied onto freshly cleaved mica and left for 15 min in a humid chamber. The substrate-deposited sample was washed twice with water and air dried. The AFM analysis was made using a scanning probe microscope NTEGRA VITA (NT-MDT Company, Zelenograd, Russia) with a standard NSG 03 (NT-MDT) silicon cantilever. The resonance frequency of the cantilever was 62–123 kHz, and its length was 100 µm. Tips with an apex curvature radius of 10 nm were used. The samples were probed in a semi-contact mode in air.

### Fibril dissociation

YB-1 (56.8 µM) was incubated with 0.15 M or 2 M KCl and 0.1 M potassium phosphate, pH 7.4, for 24 h at 37°C. An aliquot of YB-1 preincubated with 2 M KCl was diluted with water to final concentrations of 0.15 M KCl and 4.26 µM YB-1. The remaining samples were diluted to the same final protein concentration (4.26 µM) with appropriate KCl solutions to keep the salt concentration unchanged. The resulting samples were incubated for 1 h at room temperature, tested for ThT binding and imaged by EM. Alternatively, YB-1 was incubated with 0.15 M or 2 M LiCl and 20 mM Hepes-KOH, pH 7.4, for 24 h at 20°C. An aliquot of YB-1 preincubated with 2 M LiCl was dialyzed against 20 mM Hepes-KOH, 0.15 M KCl, pH 7.4. The resulting samples were imaged by AFM.

### ThT fluorescence

ThT fluorescence was analysed as described [37] with minor modifications. ThT 50×stock solution was added to a protein sample to a final ThT concentration of 50 µM. Fluorescence data were obtained using a Cary Eclipse fluorescence spectrophotometer (Varian, U.S.A.) with a 3 mm quartz cell with excitation and emission wavelengths of 440 nm and 482 nm, respectively.

### Circular dichroism spectroscopy

CD spectra were recorded using a JASCO J-810 CD spectropolarimeter (JASCO, Japan) equipped with a nitrogen-swept cell of 1 mm optical path. Samples containing 30 µM YB-1 in 20 mM Tris-HCl, pH 7.4, supplemented with either 0.15 M KCl or 1 M MgSO_4_ were analyzed. The sample with 0.15 M KCl was freshly prepared, while the sample with 1 M MgSO_4_ was incubated for 92 h at 20°C prior to analysis. Each spectrum was corrected for contributions from buffer solutions.
